# Supplementary material for: Mutations in PCYT2 disrupt etherlipid biosynthesis and cause a complex hereditary spastic paraplegia
Source: Brain. 2019 Oct 22;142(11):3382–97. doi: 10.1093/brain/awz291 (PMC6821184; doi:10.1093/brain/awz291)
Supplement: awz291_Supplementary_Data [file awz291_supplementary_data.zip › awz291-Suppl_data/Supplementary_Data4.zip!/Supplementary information 4 - plasma lipidomics.zip!/Plasma lipidomics/Control vs Patient/Statistical plots and data/Cer[d] species/Boxplots.pdf]

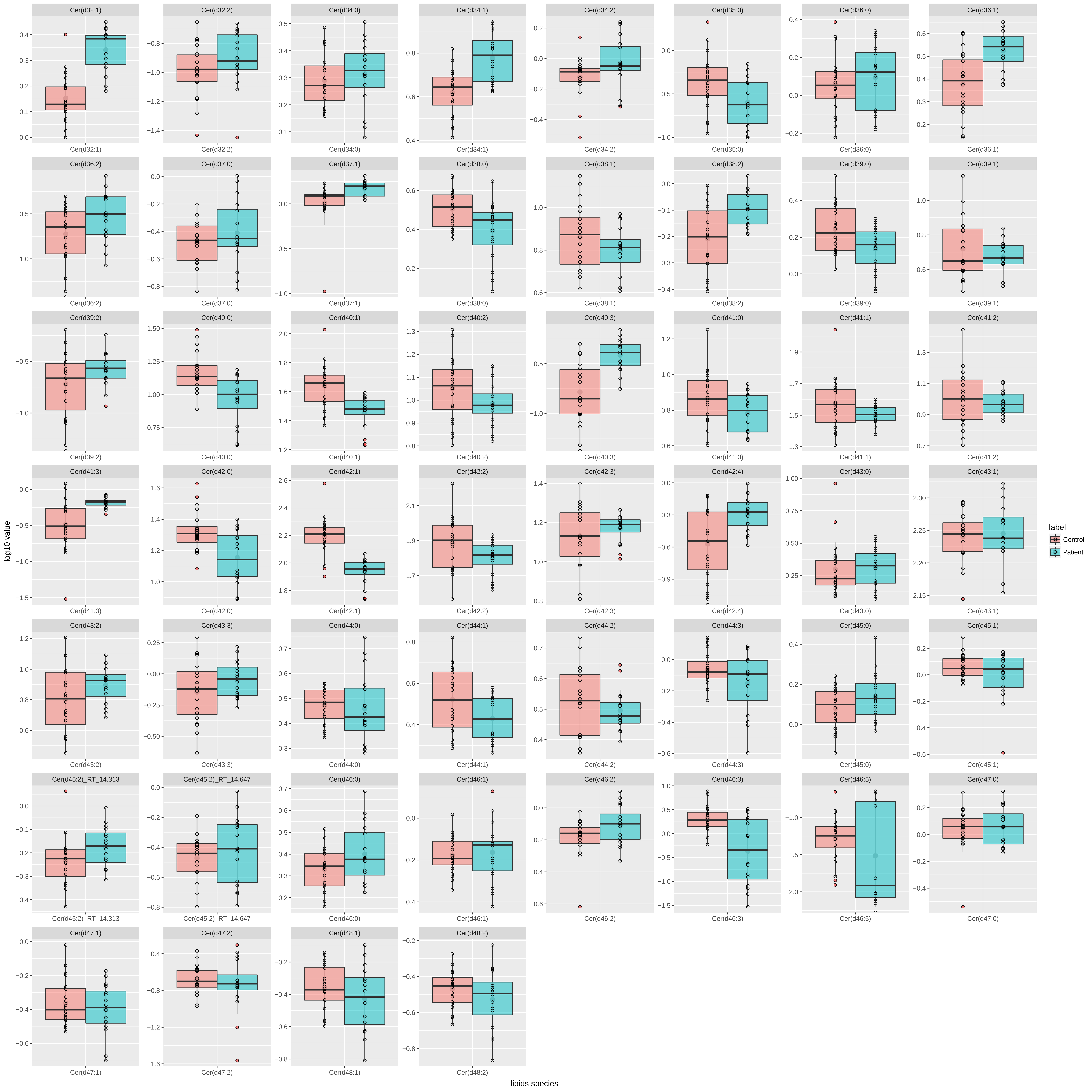
Cer(d32:1)
Cer(d32:2)
Cer(d34:0)
Cer(d34:1)
Cer(d34:2)
Cer(d35:0)
Cer(d36:0)
Cer(d36:1)
Cer(d36:2)
Cer(d37:0)
Cer(d37:1)
Cer(d38:0)
Cer(d38:1)
Cer(d38:2)
Cer(d39:0)
Cer(d39:1)
Cer(d39:2)
Cer(d40:0)
Cer(d40:1)
Cer(d40:2)
Cer(d40:3)
Cer(d41:0)
Cer(d41:1)
Cer(d41:2)
Cer(d41:3)
Cer(d42:0)
Cer(d42:1)
Cer(d42:2)
Cer(d42:3)
Cer(d42:4)
Cer(d43:0)
Cer(d43:1)
Cer(d43:2)
Cer(d43:3)
Cer(d44:0)
Cer(d44:1)
Cer(d44:2)
Cer(d44:3)
Cer(d45:0)
Cer(d45:1)
Cer(d45:2)_RT_14.313
Cer(d45:2)_RT_14.647
Cer(d46:0)
Cer(d46:1)
Cer(d46:2)
Cer(d46:3)
Cer(d46:5)
Cer(d47:0)
Cer(d47:1)
Cer(d47:2)
Cer(d48:1)
Cer(d48:2)
log10 value
lipids species
label
Control
Patient
